# Prognostic Discrimination Using a 70-Gene Signature among Patients with Estrogen Receptor-Positive Breast Cancer and an Intermediate 21-Gene Recurrence Score

**DOI:** 10.3390/ijms141223685

**Published:** 2013-12-04

**Authors:** Sung Gwe Ahn, Hak Min Lee, Hak Woo Lee, Seung Ah Lee, Se-Ra Lee, Sun-Hee Leem, Joon Jeong, In-Sun Chu

**Affiliations:** 1 Department of Surgery, Gananam Severance Hospital, Yonsei University College of Medicine, Seoul 135720, Korea; E-Mails: asg2004@yuhs.ac (S.G.A.); harumy@yuhs.ac (H.M.L.); mfreak82@nate.com (H.W.L.); 2 Department of Surgery, Eulji General Hospital, Eulji University College of Medicine, Seoul 139872, Korea; E-Mail: seungah@eulji.ac.kr; 3 Department of Biology, College of Natural Science, Dong-A University; Busan 604714, Korea; E-Mails: lsr2215@daum.net (S.-R.L.); shleem@dau.ac.kr (S.-H.L.); 4 Korean Bioinformation Center, Korea Research Institute of Bioscience and Biotechnology, Daejon 305806, Korea; E-Mail: chu@kribb.re.kr

**Keywords:** breast cancer, estrogen receptor, recurrence score, 70-gene signature, *FOXM1*, *AURKA*, *AURKB*, *BIRC5*

## Abstract

The Oncotype DX^®^ recurrence score (RS) predictor has been clinically utilized to appropriately select adjuvant chemotherapy for patients with estrogen receptor (ER)-positive early breast cancer. However, the selection of chemotherapy for patients with intermediate RSs remains controversial. We assessed the prognostic value of a 70-gene signature (70GS) among patients with ER-positive breast cancer and intermediate RSs. In addition, we sought to identify genes associated with poor 70GS scores based on gene expression profiling (GEP). GEP was performed using gene expression data from 186 patients with ER-positive breast cancer. The RS and 70GS score were calculated on the basis of GEP. Among 186 patients, 82 ER-positive patients with intermediate RSs were identified. These patients were stratified by 70GS, overall survival (OS) significantly differed according to 70GS (*p* = 0.013). In a supervised hierarchical analysis according to 70GS, the expression of several representative genes for cell proliferation was significantly higher in the poor 70GS cluster than in the good 70GS cluster. Furthermore, among these patients, *FOXM1*, *AURKA*, *AURKB*, and *BIRC5* displayed prognostic significance for OS. In conclusion, 70GS can help to discriminate survival differences among ER-positive patients with intermediate RSs. *FOXM1*, *AURKA*, *AURKB*, and *BIRC5*, are associated with poor 70GS scores.

## 1. Introduction

In estrogen receptor (ER)-positive breast cancer, which comprises approximately 75% of all breast cancers [1], endocrine therapies targeting the ER or estrogen synthesis have been the standard adjuvant therapies [2–4]. Despite the successes of endocrine therapy in reducing annual recurrences by 41% and deaths by 34%, treatment failure occurs in approximately 30% of patients treated with tamoxifen [2], and adjuvant chemotherapy has an important role for patients with a high risk of relapse. Therefore, prognosis predictors, which allow early decision making concerning appropriate adjuvant therapies in ER-positive women, have great significance in daily clinical practice.

For this purpose, the Oncotype Dx^®^ recurrence score (RS) assay, which evaluates the expression of a panel of 16 tumor-related genes and five reference genes, can be helpful in predicting the benefit of chemotherapy in patients with ER-positive breast cancer [5,6]. Specifically, the assay helps to identify patients with a poor prognosis who can derive a benefit from chemotherapy. This quantitative assay stratifies ER-positive patients into low-, intermediate-, and high-risk groups according to a numerical recurrence score. Adjuvant chemotherapy can be indicated for patients with high RSs and avoided in patients with low RSs. However, among patients with intermediate RSs, it is unclear whether the benefits of chemotherapy exceed the risks [6]. TAILORx, a large prospective study evaluating the benefits and risks of chemotherapy in these patients, had been designated to further refine the accuracy of the RS predictor [7]. This trial is closed to accrual but data collection is ongoing.

In addition to the RS predictor, several molecular predictors have been developed and clinically validated. One of these predictors is the 70-gene signature (70GS), which divides patients into those with good 70GS scores and those with poor 70GS scores [8–10]. 70GS is also commercialized and known as Mammaprint^®^. One of the differences between the RS and 70GS is that 70GS was developed in a breast cancer cohort including patients with ER-positive and ER-negative tumors.

In this study, we calculated RS and 70GS using gene expression profiling (GEP). We assessed the prognostic value of 70GS among patients with ER-positive breast cancer and intermediate RSs. In addition, we sought to discover genes associated with poor 70GS scores based on GEP.

## 2. Results and Discussion

### 2.1. Results

#### 2.1.1. Clinical Characteristics

We used a single data set of breast cancer samples from 312 women. Using exclusion criteria described in the experimental section, the clinical information of 297 patients whose tumors were donated for GEP were collected and used for analyses. ER-positive patients comprised 63% (*n* = 186) of the cohort. To classify ER-positive patients, the RS was calculated according to the algorithm presented in the experimental section. Using the RS predictor, these patients were stratified into three groups: low (*n* = 27), intermediate (*n* = 82), and high (*n* = 77). The clinical and tumor characteristics of the patients with ER-positive tumors according to the RS predictor are summarized in Table 1. The patients with high RSs exhibited higher histologic grades (*p* = 0.006), and higher proportions of progesterone receptor negativity (*p* = 0.016) and human epidermal growth factor receptor-2 (HER-2) negativity (*p* < 0.001).

**Figure 1.**
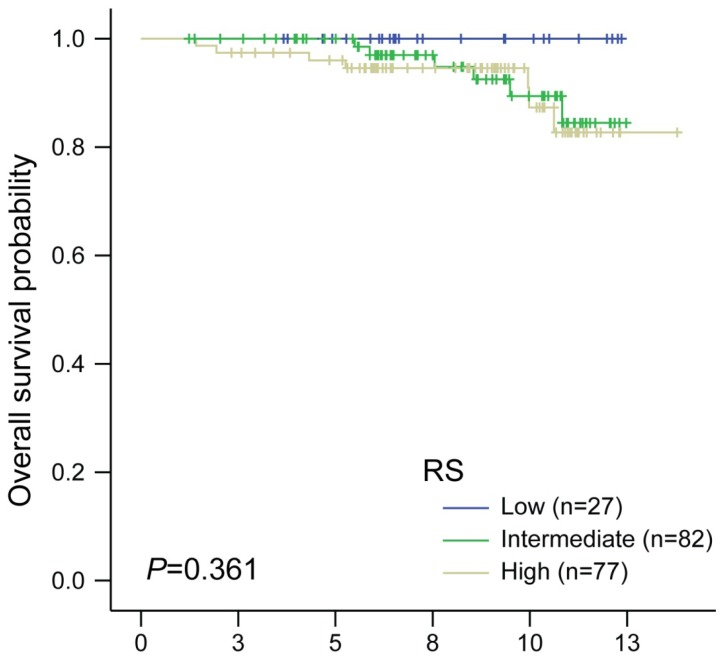
Kaplan-Meier plots for OS according to RS predictor in ER-positive patients (*p* = 0.361, log-rank test).

After a median follow-up of 8.66 years, the 10-year overall survival (OS) rate for ER-positive patients was 90.1% (95% confidence interval, 87.0–93.4). During the follow-up period, 12 breast cancer specific-mortalities and one non-breast cancer specific-mortality occurred. No mortalities occurred among patients with low RSs. In the patients with intermediate and high RSs, five and six mortalities were observed, respectively. A Kaplan-Meier plot for OS according to the RS predictor is illustrated in Figure 1 (*p* = 0.361, log-rank test).

#### 2.1.2. Survival Analysis Using 70GS in ER-Positive Patients with Intermediate RSs

**Figure 2.**
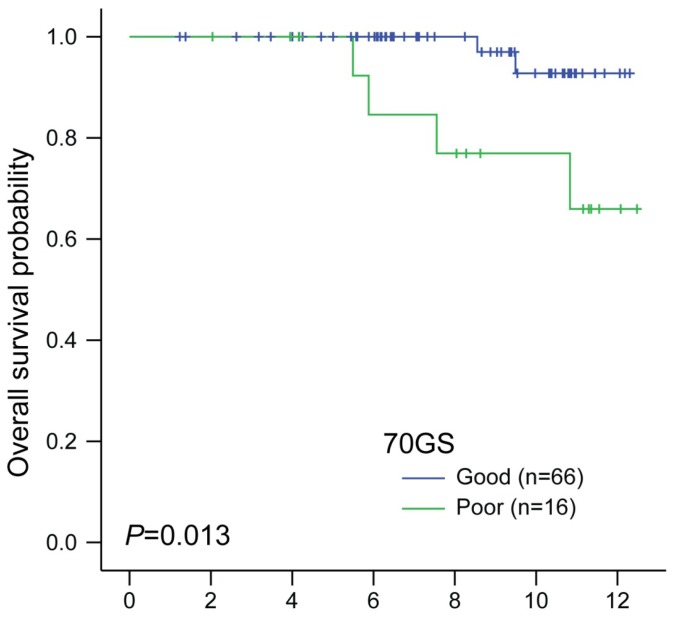
Kaplan-Meier plots for OS according to 70GS predictor in ER-positive patients with intermediate RSs (*p* = 0.013, log-rank test).

We further analyzed ER-positive patients with intermittent RSs (*n* = 82). Survival analysis using 70GS was performed in this group. The 70GS score was also calculated according to the algorithm described in the experimental section. The 70GS score stratified these patients into two groups: good (*n* = 66) and poor (*n* = 16). Baseline characteristics are presented in Table 2. The patients with poor 70GS scores had larger tumor sizes and higher histologic grades (*p* = 0.040 and *p* = 0.034, respectively). In a survival analysis using the log-rank test, OS significantly differed according to the 70GS predictor (*p* = 0.013, Figure 2). In the univariate analysis using other characteristics, receipt of adjuvant chemotherapy was only analyzed as a significant factor (*p* = 0.024; Table S1). In multivariate analysis using Cox regression hazard model, poor 70GS (adjusted HR 10.19) was demonstrated as an independent prognostic factor for OS, whereas non-receipt of adjuvant chemotherapy (adjusted HR 64.18) was also associated with an increased risk of mortality (Table 3). For this model, Harrell *c*-index was 0.844. From this analysis, we confirmed the prognostic ability of 70GS in this subset.

#### 2.1.3. Supervised Hierarchical Clustering Analysis According to 70GS

**Figure 3.**
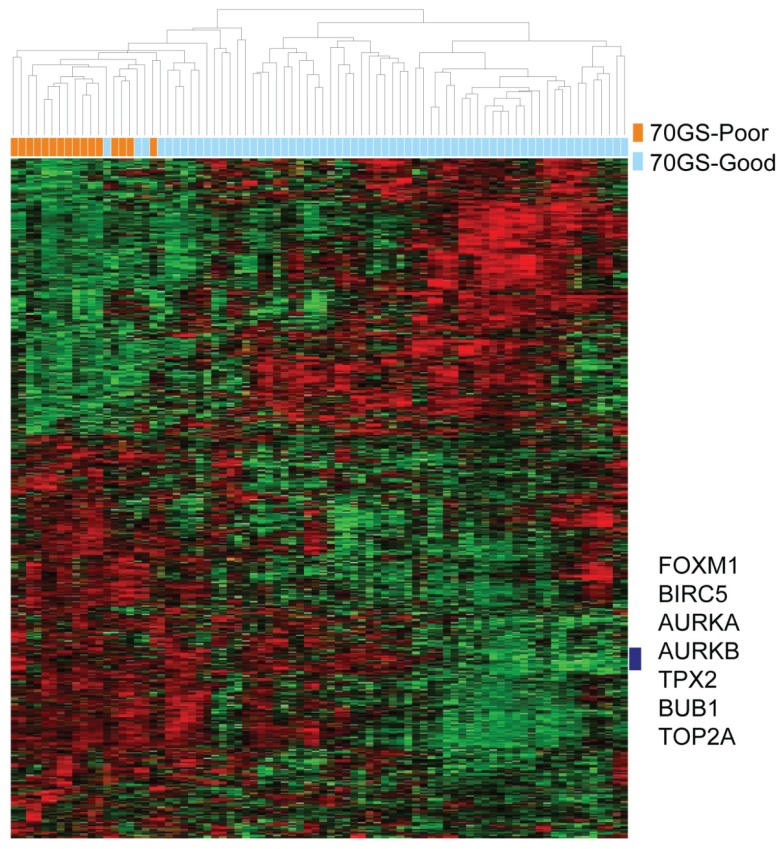
Hierarchical clustering analysis of 487 gene features according to 70GS among patients with ER-positive breast cancer and intermediate risk scores. Columns represent individual samples, and rows represent individual genes. Red and green reflect high and low expression levels (log2-transformed scale), respectively.

Next, to identify genes associated with poor 70GS scores, supervised hierarchical clustering analysis, according to 70GS, was performed using 82 tumor samples. Genes with expression levels that differed by at least two-fold in at least eight tissues relative to the median value across tissues were selected. Among genes expressed in the Illumina BeadArray, we selected genes with significantly differences in expression between the poor and good 70GS clusters using Student’s *t*-test (*p* < 0.01). We identified 487 gene features that were differentially expressed between the two clusters based on an average linkage clustering method (Figure 3, Table S2). We further investigated overexpressed genes in the poor 70GS cluster. In this study, under-expressed genes in the poor 70GS cluster were not evaluated. To identify the favored genes in the poor 70GS cluster, we used Ingenuity Pathway Analysis™ Tool. In addition, genes showing at least two-fold change between two clusters were explored by review of references. In this way, among highly expressed genes in the poor 70GS cluster, *FOXM1*, *AURKA*, *AURKB*, *BIRC5*, *BUB1*, and *TOP2A*, were identified, and the prognostic significance of these genetic markers was analyzed. These genes, which are mainly associated with cell cycle progression (*FOXM1*), mitosis (*AURKA*, *AURKB*, *BUB1*), apoptosis (*BIRC5*), DNA transcription and replication (*TOP2A*), were significantly higher in the poor 70GS cluster than in the good 70GS cluster.

#### 2.1.4. Identification of Genetic Markers Associated with a Poor Prognosis in ER-Positive Patients with Intermediate RSs

**Figure 4.**
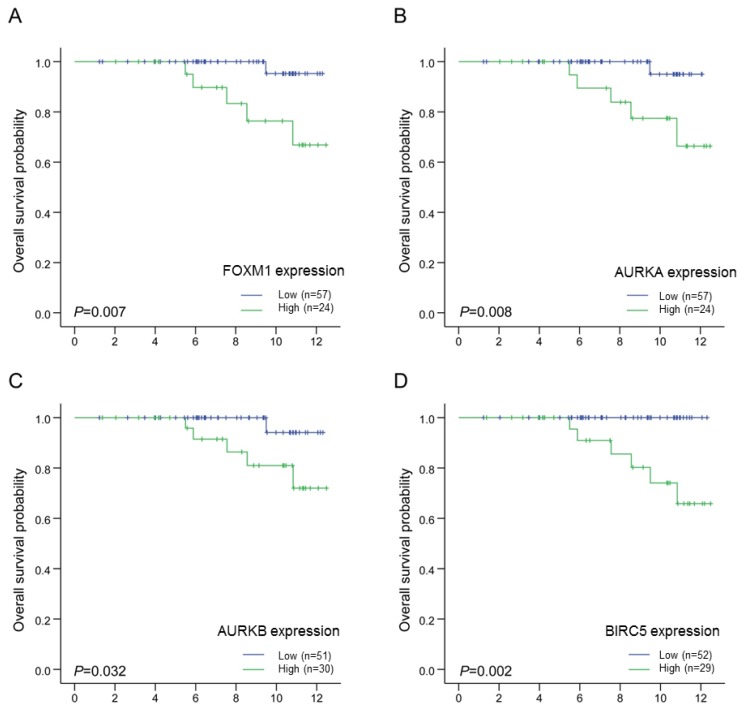
Kaplan-Meier plots for OS according to each genetic marker in ER-positive patients with intermediate RSs. All *p*-values were measured by the log-rank test. (**A**) *FOXM1*, *p* = 0.007; (**B**) *AURKA*, *p* = 0.008; (**C**) *AURKB*, *p* = 0.032; (**D**) *BIRC5*, *p* = 0.002.

Following the identification of genetic markers related with poor 70GS scores, we then proceeded to perform survival analysis using these markers in ER-positive patients with intermediate RSs. Among 82 patients, the expression levels of these genetic markers were confirmed in 81 women. The cutoffs of each marker were determined as the median values of the expression scores. Using the log-rank test, we investigated the clinical significance of these markers for OS in this subset. We discovered that *FOXM1*, *AURKA*, *AURKB*, and *BIRC5* were associated with a poor prognosis in ER-positive patients with intermediate RSs (*p* = 0.007, *p* = 0.008, *p* = 0.032, and *p* = 0.002, respectively; Figure 4). The other two genes were not demonstrated to be associated with a poor prognosis in these patients (BUB1, *p* = 0.570; TOP2A, *p* = 0.185). Harrell’s *c*-indices, in which increasing values between 0.5 and 1.0 indicate improved prediction, were calculated for OS for each significant marker. These values were 0.796 for *FOXM1*, 0.792 for AURKA, 0.755 for AURKB, and 0.821 for BIRC5. Additionally, these proliferative gene markers displayed 72%–85% agreement with 70GS (*FOXM1*, 85%; *AURKA*, 80%; *AURKB*, 75%; *BIRC5*, 72%; Table 4).

### 2.2. Discussion

The RS predictor, which is based on the expression of 21 genes using reverse transcriptase-polymerase chain reaction in formalin-fixed, paraffin-embedded tissue, has been widely adopted in clinical practice for early decision making concerning adjuvant therapy in patients with ER-positive breast cancer. The RS predictor has prevented the unnecessary use of adjuvant chemotherapy in a large proportion of patients with ER-positive breast cancer. However, in patients with intermediate RSs, the RS predictor remains questionable for selecting adjuvant treatment. To fulfill an unmet need for this group, the TAILORx trial, which is randomizing patients with intermediate RSs to receive either hormonal therapy alone or hormonal therapy and chemotherapy, was launched and closed to recruitment of patients [7].

In this study, we showed that 70GS can identify a survival difference among ER-positive patients with intermediate RSs, who the TAILORx trial is primarily targeting. It is interesting finding that 70GS can provide prognostic information for patients in the gray zone according to the RS predictor. 70GS has been validated as a significant prognosticator in various cohorts, including node-negative [11], node-positive [12], postmenopausal [13], metastatic [14], and neoadjuvant cohorts [15]. This predictor is used to avoid unnecessary adjuvant systemic treatment in patients with good gene signatures. Regarding this issue, MINDACT trial is prospectively validating 70GS in the patients with N0–N1 tumors [16].

In supervised hierarchical analysis according to 70GS, we discovered 487 genes that were differently expressed between two clusters. Among these genes, we identified enriched gene signatures related to cell proliferation in the poor 70GS cluster. Further, we showed the prognostic value of four genes. One of the genes, *FOXM1*, is an oncogenic transcription factor of the Forkhead family, and it has a master role in cell proliferation and cell cycle progression [17–19]. In quiescent or differentiated cells, *FOXM1* is expressed at a lower level, whereas *FOXM1* is consistently detected in highly proliferating cells [20]. High *FOXM1* expression has been found in various human malignancies [21], and it also correlates with poor prognosis in patients with breast cancer [22]. Increased *FOXM1* transcriptional activity can stimulate genes involved in diverse hallmarks of cancer and contribute to tumorigenesis [18]. Recently, scientists with The Cancer Genome Atlas also emphasized that hyperactivated *FOXM1* as a transcriptional driver of enhanced proliferation signatures is important in differentiating between luminal A and luminal B tumors [23]. Therefore, *FOXM1* can provide additional prognostic information, even in ER-positive tumors with intermediate RSs.

*AURKB* is also related to *FOXM1* proliferation gene signatures. *AURKB* is directly upregulated by activated *FOXM1*, which results in a malignant change of cell phenotype [24]. The other two genes, *AURKA* and *BIRC5*, were originally categorized as proliferative genes in the RS predictor [5]. Our data imply that these two genes play an important role in predicting prognosis using the RS predictor for patients with intermediate RSs.

Similar gene signatures as observed in our results are also in the gene expression grade index (GGI) developed by Sotiriou *et al*. [25]. The GGI was developed to reclassify patients with histologic grade 2 tumors into two different prognostic groups. Most of these genes in the GGI are involved in cell cycle regulation and proliferation, and it is noteworthy that *FOXM1*, *AURKA*, and *BIRC5* are among the top 20 overexpressed genes in the GGI [25].

Among several molecular predictors developed for predicting prognosis or early decision making concerning adjuvant chemotherapy among patients with ER-positive breast cancer, key genes are commonly categorized as proliferation genes. Several papers demonstrated that these proliferation genes can predict outcome [25,26]. In an era of tailored medicine, based upon molecular analyses, better tools to accurately categorize these tumors will be anticipated in clinical practice. Thereby, proliferation genes including *FOXM1* and associated gene signatures may become more important markers in future discrimination.

Interestingly, in the multivariate analysis, we found that adjuvant chemotherapy significantly affected overall survival in patients with intermediate RSs. During the study period, our patients received adjuvant chemotherapy according to the St. Gallen’s guideline. Thus, this finding does not allow to draw the conclusion that adjuvant chemotherapy might offer a survival benefit for patients with intermediate RSs.

Our study possesses several caveats. The retrospective design is associated with inherent limitations. Our study, which is not based on clinical trials, could not control for individual variations in adjuvant therapy that influenced survival. In addition, our 21-gene recurrence scores were not directly compared with real OncotypeDx^®^ assays.

Despite the limitations of the retrospective design and uncontrolled adjuvant treatments, our results provided relevant evidence to be considered in ER-positive patients with intermediate RSs.

## 3. Experimental Section

### 3.1. Tumor Samples Used for Gene Expression Profiling

We used a single data set of breast cancer samples from 312 women. We prospectively collected tumor tissues from specimens of surgically resected breast carcinoma at the Gangnam Severance Hospital, Yonsei University College of Medicine, Seoul, Korea between July, 1997 and December, 2007. Following exclusion criteria, 297 patients with invasive breast carcinoma were finally utilized for GEP. Patients with pure *in situ* carcinoma of the breast, recurrent or metastatic disease, bilateral breast cancers, or nonepithelial origin breast cancer, such as phyllodes tumor, sarcoma, or lymphoma, as well as those receiving neoadjuvant chemotherapy, were excluded. The clinical data of the patients, including age, tumor size, histologic grade, lymph node status, and the expression status of ER, PR, and HER-2, were retrieved from the database. TNM disease stage was classified according to the American Joint Committee on Cancer staging manual, 7th edition. The modified Scarf-Bloom-Richardson grading system was used for tumor grading. HER-2 positivity was assessed by three positive results on immunohistochemistry or fluorescence *in situ* hybridization amplification.

### 3.2. Ethics Statement

The institutional review board of Gangnam Severance Hospital, Yonsei University, Seoul, Korea, approved the study in accordance with good clinical practice guidelines and the Declaration of Helsinki. The requirement for informed consent was waived because of the retrospective design.

### 3.3. RNA Extraction, Microarray Experiments, and Data Processing

Total RNA was isolated from the tissues with Trizol (Life Technologies, Carlsbad, CA, USA) reagent according to the manufacturer’s protocol. Five hundred nanograms of total RNA were used for labeling and hybridization according to the manufacturer’s protocols (Illumina, San Diego, CA, USA). The quality of the RNA obtained from each tumor sample was assessed via the RNA profile generated. Samples with a total area under the 28S and 18S bands of less than 15% of the total RNA band area, as well as a 28S/18S ratio of less than 1.1, were degraded and not analyzed further (approximately 20% of the samples). Only tumor samples with good-quality RNA were considered for further analysis. RNA amplification, hybridization, and scanning were performed. After the bead chips were scanned with an Illumina BeadArray Reader (San Diego, CA, USA), the microarray data were normalized using the quantile normalization method in the Linear Models for Microarray Data package in the R language environment. The default options of RMA (with background correction, quantile normalization, and log transformation) were used. Microarray study was performed by the Shared Research Equipment Assistance Program by Korea Basic Science Institute, MEST.

### 3.4. Multigene Assay Calculation

To classify tumors using the RS predictor, we used the microarray data for all 21 RS genes and applied the algorithm and scaling methods described by Paik *et al*. [5]. First, the 21 genes were identified on the current platform using gene symbols and a single probe set was selected as representative expression measure. Next, the expression levels of the 16 prognostic genes were normalized by dividing these with the mean expression values of the five reference genes (*ACTB*, *TFRC*, *RLPL0*, *GUS*, and *GAPDH*). The normalized expression levels of each prognostic gene were then scaled by subtracting the minimum expression values across all 16 genes as in the original publication. The reference normalized- and scaled-expression values ranged from 0 to 8.25 on log2-scale and were used to calculate gene group scores. Group scores were generated by multiplying the expression level of a gene by the pre-determined constant of the gene group according to the original published algorithm and a raw recurrence score was calculated as RS = 0.47 × GRB7 group score −0.34 × ER group score + 1.04 × proliferation group score + 0.1 × invasion group score [5]. This raw score was rescaled by multiplying it with 20 and subtracting the minimum score as in the original manuscript [5]. Using the cutoffs described by Paik *et al*. (0–18, 19–30, 31–100), we assigned each patient into a low-, intermediate-, or high-risk group.

For the 70-gene prediction, genes in 70GS were identified on the Illumina platform using gene symbols, and their expression data were centralized across the patients before calculation of the prognostic index. Each gene was represented by a single probe set and when multiple probes targeted the same gene, probes with the highest variance were selected 70GS was calculated as the weighted sum of the gene-expression values. The weight was calculated as the expression level of a gene multiplied by its predetermined correlation coefficient that was taken from the original publication [9,10]. Patients with a correlation coefficient >0.4 were classified as good, and whereas those with coefficients ≤0.4 were categorized as poor [9,10,27].

### 3.5. Statistical Analysis

To visualize gene expression values using heat maps, the values for each probe were centered by subtracting the mean expression value across patients. No gene-specific scaling (standardization) was performed, and, thus, information about the relative signal strength between probes was retained. The color tone in the heat maps was calibrated so that saturated red and saturated green were reached at values equal to three-fold the standard deviation of the expression values of the entire matrix. Red and green reflect high and low expression levels (log2-transformed scale), respectively.

Age is presented in the study as the median value and range and compared using the Mann-Whitney *U* test. Other discrete variables were compared using the chi-square test. OS was measured from the date of the first curative surgery to the date of the last follow-up or until death from any cause during follow-up. The Kaplan-Meier method was utilized to estimate OS, and the estimated survival curves were compared using the log-lank test. The significant prognostic factors associated with disease-free survival were selected using Harrell *c*-statistic [28] and a Cox proportional hazard regression model was applied for multivariate survival analysis. Harrell’s *c*-statistic was also used to identify the predictive ability for OS in each genetic marker. SPSS version 18 (SPSS; Chicago, IL, USA) and R (http://www.r-projet.org) were used to perform these analyses. Statistical significance was defined by *p* < 0.05.

## 4. Conclusions

70GS can discriminate survival differences among ER-positive patients with intermediate RSs. Proliferative gene signatures including those of *FOXM1*, *AURKA*, *AURKB*, and *BIRC5* are associated with poor 70GS scores.

## Supplementary Information



## Figures and Tables

**Table 1.** Baseline characteristics according to the recurrence score (RS) predictor in estrogen receptor (ER)-positive patients (*n* = 186).

Characteristics	Low (*n* = 27)	Intermediate (*n* = 82)	High (*n* = 77)	*p*-value
Age (median, range)	48 (33–72)	45 (25–74)	47 (22–69)	0.868
Tumor size				0.099
*T* ≤ 2 cm	15	34	25	
*T* > 2 cm	12	48	52	
Nodal status				0.383
Negative	11	46	40	
Positive	16	36	37	
Stage				0.221
I	9	24	19	
II	10	47	40	
III	8	11	18	
Histologic grade				0.006
I, II	25	68	50	
III	1	7	17	
Unknown	1	7	10	
Progesterone receptor				0.016
Positive	25	74	58	
Negative	2	8	19	
HER-2 *				<0.001
Positive	0	4	18	
Negative	24	59	46	
Unknown	3	19	13	
Adjuvant chemotherapy				0.089
Yes	22	68	72	
No	5	14	6	
Adjuvant endocrine therapy				0.760
Yes	24	68	65	
No	3	14	12	
Adjuvant radiotherapy				0.107
Yes	10	31	41	
No	17	51	36	

HER-2, human epidermal growth factor receptor-2;

* HER2 positivity was defined by three positive findings in an immunohistochemical examination or amplification in fluorescence *in situ* hybridization.

**Table 2.** Baseline characteristics according to 70-gene signature (70GS) predictor in ER-positive patients with intermediate RSs (*n* = 82).

Characteristics	Good (*n* = 66)	Poor (*n* = 16)	*p*-value
Age (median, range)	44 (28–62)	48 (22–69)	0.059
Tumor size			0.040
*T* ≤ 2 cm	31	3	
*T* > 2 cm	35	13	
Nodal status			0.989
Negative	37	9	
Positive	29	7	
Stage			0.059
I	21	3	
II	9	8	
III	6	15	
Histologic grade			0.034
I, II	56	12	
III	3	4	
Unknown	7	0	
Progesterone receptor			0.650
Positive	60	14	
Negative	6	2	
HER-2 *			0.754
Positive	3	1	
Negative	40	11	
Unknown	15	4	
Adjuvant chemotherapy			0.200
Yes	53	15	
No	13	1	
Adjuvant endocrine therapy			0.348
Yes	56	12	
No	10	4	
Adjuvant radiotherapy			0.978
Yes	25	6	
No	41	10	

HER-2, human epidermal growth factor receptor-2;

* HER2 positivity was defined by three positive findings in an immunohistochemical examination or amplification in fluorescence *in situ* hybridization.

**Table 3.** Multivariate analysis using the log-rank test according to the characteristics.

Characteristics	*p*-value	HR (95% CI)
**Tumor size**	0.115	11.94 (0.55–260.07)
*T* ≤ 2 cm *vs. T* > 2 cm		
**Nodal status**	0.987	1.91 (0.26–13.99)
Negative *vs*. Positive		
**Progesterone receptor**	0.525	1.03 (0.04–27.97)
Positive *vs*. Negative		
**70GS**	0.045	10.19 (1.05–99.01)
Good *vs*. Poor		
**Adjuvant chemotherapy**	0.013	64.18 (2.44–1685.7)
Yes or No		

**Table 4.** Agreement between each genetic marker and 70GS.

		FOXM1		AURKA		AURKB		BIRC5	
		
		Low (*n* = 57)	High (*n* = 24)	Low (*n* = 57)	High (*n* = 24)	Low (*n* = 51)	High (*n* = 30)	Low (*n* = 52)	High (*n* = 29)
70GS	Good (*n* = 65)	55	10	53	12	48	17	47	18
	Poor (*n* = 16)	2	14	4	12	3	13	5	11
