# Supplementary material for: Prognostic Discrimination Using a 70-Gene Signature among Patients with Estrogen Receptor-Positive Breast Cancer and an Intermediate 21-Gene Recurrence Score
Source: Int J Mol Sci. 2013 Dec 4;14(12):23685–99. doi: 10.3390/ijms141223685 (PMC3876071; doi:10.3390/ijms141223685)
Supplement: Supplementary file 1 [file ijms-14-23685-s001.pdf]

Supplementary Information

Table S1. Univariate analysis using the log-rank test according to the characteristics.

	***p*-value**
**Tumor size**	0.758
T ≤ 2 cm *vs*. T > 2 cm	
**Nodal status**	0.825
Negative *vs*. Positive	
**Stage**	0.371
I *vs*. II *vs*. III	
**Histologic grade**	0.442
I-II *vs*. III	
**Progesterone receptor**	0.754
Positive *vs*. Negative	
**HER-2 ***	0.756
Negative *vs*. Positive	
**70GS**	0.013
Good *vs*. Poor	
**Adjuvant chemotherapy**	0.024
Yes or No	
**Adjuvant endocrine therapy**	0.811
Yes or No	
**Adjuvant radiotherapy**	0.085
Yes or No	

HER-2, human epidermal growth factor receptor-2; ***** HER2 positivity was defined by three positive findings in an immunohistochemical examination or amplification in fluorescence *in situ* hybridization.

© 2013 by the authors; licensee MDPI, Basel, Switzerland. This article is an open access article distributed under the terms and conditions of the Creative Commons Attribution license (http://creativecommons.org/licenses/by/3.0/).
